# Usability and Acceptability of a Digital Health Activity Program among Older Heart Failure Family Care Partners

**DOI:** 10.1093/geroni/igaf122.3469

**Published:** 2025-12-31

**Authors:** Dawon Baik, Catherine Jankowski, Larry Allen, Colleen McIlvennan, Heather Coats, Blaine Reeder

**Affiliations:** University of Colorado, Aurora, Colorado, United States; University of Colorado, Aurora, Colorado, United States; University of Colorado, Aurora, Colorado, United States; University of Colorado, Aurora, Colorado, United States; University of Colorado, Aurora, Colorado, United States; University of Missouri, Columbia, Missouri, United States

## Abstract

Family care partners of persons with heart failure (HF-FCPs) face significant stress and exhaustion from prolonged caregiving, impacting their health. As they age, their increased burden and limited support raise the risk of poor health outcomes for both themselves and the care recipients. To support older HF-FCPs’ health management, we developed a tailored digital health physical activity (PA) program, called TPA4You, that includes videoconferencing with a health coach, wearable sensors, and motivational messaging. We conducted a 1-week, two-phase, iterative field test with 11 participants to evaluate the usability and acceptability of TPA4You. Modifications were made based on Phase 1 participants’ feedback (i.e., reducing weekly PA sessions from three to two, replacing the automated messaging system with manually sent motivational messages). All participants in both phases (mean age 68.2±7.1 years, 91% female, 82% spouses) attended 100% of Zoom PA sessions. Key themes from Phases 1 and 2 were similar. Overall, participants found TPA4You acceptable and useful. One key theme was TP4AYou is a digital tool that builds confidence in PA capabilities by increasing awareness and motivation. Another key theme was TPA4You’s ability to provide emotional and psychosocial benefits by reducing caregiver stress and facilitating human contact with a health coach. A third theme was TPA4You served as a valuable reminder to take care of themselves. In Phase 2, manual motivational messages led to higher satisfaction, less confusion, and greater PA engagement as compared to Phase 1 automated messaging. These findings will inform further revisions and a pilot RCT of TPA4You.

